# Clinical Profile of Dengue Fever in Children: A Study from Southern Odisha, India

**DOI:** 10.1155/2016/6391594

**Published:** 2016-04-24

**Authors:** Shubhankar Mishra, Ramya Ramanathan, Sunil Kumar Agarwalla

**Affiliations:** Department of Paediatrics, MKCG Medical College, Berhampur, Odisha 760004, India

## Abstract

*Background*. In India, dengue epidemics are becoming more frequent (WHO, 2008). The majority of dengue viral infections are self-limiting, but complications may cause high morbidity and mortality.* Objectives*. To assess the clinical profile of the dengue infection in children less than 14 years of age and to evaluate the outcomes of dengue fever from September 2013 to August 2015 at the Pediatric Department of Maharaja Krishna Chandra Gajapati Medical College, the largest tertiary care hospital of southern Odisha.* Results*. A total of 97 cases were classified into 84 (86.59%) nonsevere and 13 (13.40%) severe dengue cases. The most common age of presentation was above 11 yrs. The mean age of admission was 8.7 yrs. The most common presenting symptom was fever seen in 100% and hepatomegaly (43.8%), the most common physical finding. Gastrointestinal bleeding was markedly seen in severe dengue (76.9%). Elevation in aspartate transaminase (SGOT) was found in 47.42% and thrombocytopenia in 27.5%. The correlation between hepatomegaly and elevated SGOT was significant (*P* value 0.0346). Case fatality rate (CFR) was 1.03%. The mean duration of hospitalisation was 3.8 days.* Conclusion*. In children, if symptoms like fever, pain, rashes, and vomiting are associated with hepatomegaly and elevated SGOT in context of low TPC, a strong possibility of dengue fever is present, especially in an epidemic setting. Early suspicion and effective management can reduce the severity.

## 1. Introduction

Globally 50 million dengue infections are reported annually [1]. The first dengue fever in India was reported during 1956 from Vellore and the first dengue haemorrhagic fever occurred in Calcutta in 1963 [2]. In India the annual incidence is estimated to be 7.5 to 32.5 million [3]. In Odisha, a state of Eastern India, the first outbreak was reported in 2010, followed by extensive outbreaks in 2011, affecting a large number of people. According to the WHO the case fatality rate for dengue is roughly 5% [1].* Aedes albopictus* was found to be the most abundant vector in the areas surveyed, followed by* Aedes aegypti*. DENV-2 is the prevailing serotype [4]. The case fatality rate in patients with severe dengue infection which consists of dengue haemorrhagic fever (DHF) and dengue shock syndrome (DSS) can be as high as 44% [2, 5]. If intervention occurs early, mortality is less than 1% [6]. Dengue fever presents as common viral fever which causes dangerous complications. Dengue reinfection is observed to be more severe in children due to immunological phenomenon [7]. In 2010, 25 cases and five deaths were reported from Odisha [8]. Rapid increase in the dengue cases in 2012 became a public health concern in Eastern India as the majority of cases were affecting the young adolescents. The objective of this study was to assess the clinical profile of the dengue infection in children less than 14 years of age and to evaluate the outcome of dengue fever in the southeastern part of India where dengue outbreaks are rampant.

## 2. Material and Methods

It was a prospective observational study. All probable cases that had high grade fever, lymphadenopathy, hepatomegaly, features of shock or haemorrhage, and so forth and were admitted with provisional diagnosis of dengue fever to the Paediatric Ward of Maharaja Krishna Chandra Gajapati Medical College, Berhampur, Odisha, were taken into account. All children aged up to 14 years with positive dengue tests, either NS1 antigen, IgM, IgG antibody rapid serological test kit (J. Mitra and Co. Pvt. Ltd.), or ELISA, were taken into the study group. As the duration of history of fever might be fallacious the patients were subjected to all three serological tests. Children who were positive for malaria, meningitis, and enteric fever were excluded from the study. The whole number of patients included in our study was 97 (*n* = 97). Cases were followed up daily for the clinical and laboratory parameters. Blood parameters were monitored every day till remarkable improvement seen clinically and haematologically. Averages of TLC, TPC, Hb, haematocrit, and so forth were calculated for each patient and recorded. Daily vitals were monitored with tourniquet test. Chest X-ray, ultrasonography, and liver function tests were done on day 3 of admission on all the patients. The patients were treated with oral paracetamol, intravenous fluids, blood products, and inotropes as per the recent WHO dengue guidelines [2]. The frequency of various signs and symptoms and the laboratory tests were compared between the nonsevere and severe disease. The results were tabulated and correlated. The outcomes were recorded.

The clinical manifestations and laboratory findings like haemoglobin estimation, total platelet count, haematocrit estimation, NS1 antigen, and IgM antibody of each group of illness were compared using * Fisher's exact test* for proportions. GraphPad version 6.0. software and SPSS version 22.0.0.0 software were used for data entry and analysis. *P* value below 0.05 was considered significant. Written consent was taken from the parents before enrolling in the study. Ethical committee clearance was taken from Maharaja Krishna Chandra Gajapati Medical College before starting this study.

## 3. Observations and Results

The total number of cases was 97, out of which 84 were cases of nonsevere dengue (undifferentiated fever, dengue fever with warning signs, and dengue fever without warning signs) and 13 were cases of severe dengue (DHF and DSS) according to WHO guidelines [2]. There were 75 (77.31%) males and 22 (22.68%) females in our study. Both the groups of severe and nonsevere dengue males had high incidence. The male-to-female ratio was 3.4 : 1. The maximum number of cases, 33 (34.02%), was seen in the group above 11 years of age, out of whom male children were 25 (33.33%) and female children 8 (36.36%). The mean age of hospitalised patients was 8.7 yrs. 63.9% of patients were admitted in the hospital for 3–6 days. Seven children out of 13 severe dengue patients were admitted for more than 6 days. The mean tenure of hospitalisation was 3.8 days. In severe dengue cohort the mean stay was 5.8 days. The mean delay in admission after appearance of fever was 3.4 days (Table 1). The total admissions for diagnosed cases of dengue in adults were 211 in our hospital in the duration of time of our study.

The majority of the cases were admitted in the rainy and winter season between the months of July and November. The peak of admission was seen in the month of September with 59 cases (60.8%). The least admission was seen in the summer season, specifically in the month of April, consisting of 4 cases (4.1%).

Fever was present in 100% of the cases; myalgia (76.8%) and abdominal pain (54.3%) were common. Hepatomegaly (43.8%) was the most common physical finding. The most common bleeding manifestations in both severe and nonsevere dengue were petechiae (22.1%). Gastrointestinal bleeding was significantly seen in severe dengue cases. 58.76% of the cases had normal leukocyte count, while leucopoenia was seen in 25.77% and leukocytosis in 15.46% of the cases. Among the liver enzymes, SGOT was elevated in a larger proportion (47.42%) of patients when compared to alanine aminotransferase (SGPT) which was 30.92%. Elevation in SGOT was significantly seen in those with severe dengue (76.92%, *P* value: 0.0346) rather than nonsevere dengue (42.85%). SGPT was very high (>1000 IU/L) in 3 patients whereas SGOT was very high (>1000 IU/L) in 5 patients. All these patients with high liver enzymes had other severities also. Out of them one child that had very high titre of both liver enzymes succumbed despite admission to the intensive care unit. Parameters like prothrombin time (PT) and activated partial thromboplastin time (aPTT) were abnormal in 23 (23.7%) patients. In 10 (76.9%) cases of severe dengue PT/aPTT levels came out to be abnormal. 27.83% presented with thrombocytopenia (platelet < 100000). 69.23% of severe dengue cases had thrombocytopenia whereas only 21.42% of nonsevere dengue cases had thrombocytopenia. Thrombocytopenia was seen to be more relevant in those with severe dengue. One of the important findings of dengue is raised haematocrit which was seen in 34.02% of the cases and it was statistically not significant.

25.77% of the cases were detected to have pleural effusion by chest X-ray (PA view and oblique view in right lateral decubitus). Right sided effusion (15.46%) was most commonly seen followed by bilateral effusion (6.18%). Among the severe dengue cases, the majority, 38.46%, presented with bilateral effusion. Ultrasound of the abdomen detected hepatomegaly in 52.75% of the cases, which is the most common finding followed by ascites (25.77%) and gall bladder wall edema (2.06%).

In our study, the majority of the patients were positive for NS1 followed by IgM (Table 2) as a large number of patients presented within 4 days of fever. Serum IgG was estimated in those children who presented with history of 7 days. Tourniquet test was found to be negative in the majority of the patients. In this study it was found that the bleeding manifestation had no correlation with thrombocytopenia, hepatomegaly, and raised SGOT (Table 3). All 97 patients had fever and they were treated with antipyretics (paracetamol) in appropriate doses. Patients who presented with warning signs and stable vital signs were initially encouraged to take oral fluids; if they were not tolerated intravenous fluids were started according to the WHO guidelines. Among 97 patients, 27.83% of the cases needed intravenous fluids and the majority were severe dengue cases (76.92%). Dopamine was required in 4.12% of the cases and all of them were severe dengue cases (30.76%). Adrenaline was used only in one patient who belonged to the severe dengue category. Platelet concentrate was given for 4 severe dengue cases. Whole fresh blood transfusion was needed in 4.12% of the cases and all of them were severe dengue cases (Table 4). In our study all 84 (86.59%) of nonsevere dengue cases recovered. Among the severe dengue cases, 13 (13.40%), 12 cases (12.37%) recovered and 1 case (1.03%) expired due to intractable shock.

## 4. Discussion

Dengue is an important arboviral infection in tropical countries. Global incidence of dengue fever has increased dramatically in the recent decades [6]. There are very few studies based on the revised new dengue classification.

Based on the WHO TDR 2009 dengue guidelines, in our study, the total number of cases analysed was 97, out of which 84 (86.59%) were categorised as cases of nonsevere dengue which included both undifferentiated fever and dengue fever (DF) (both with and without warning signs) and 13 (13.40%) were cases of severe dengue (DHF grades 1–4). The maximum numbers of cases were seen in the group >11 years of age (34.02%) and the least affected age group was infants. More involvement in adolescent children can be explained by diurnal adaptation of* Aedes* mosquito in stored water. These children work in open field. This makes them prone to repeated attacks by* Aedes* mosquitoes. There was significant difference in male : female ratio in our study (3.4 : 1) whereas in other studies there were no such significant differences [9]. This was probably due to more importance being given to the male children in the Indian society. Covered dress used by females may be another cause for fewer incidences. Increased admissions in the rainy and winter seasons can be explained by breeding season of mosquitoes which is similar to previous studies [10]. Duration of hospitalisation was more in case of severe dengue patients. But delay in hospitalisation did not predict the severity in our study.

In our study fever was present in all cases. Abdominal pain, vomiting, retroorbital pain, and abdominal distension were seen commonly. This goes with previous study [10]. Bleeding in dengue is multifactorial. The most common bleeding manifestations in both severe and nonsevere dengue were petechiae, purpura, and ecchymosis. Gastrointestinal bleeding was significantly seen in severe dengue cases. Haematemesis was the most common bleeding manifestation reported in other Indian studies. Convulsion due to infection is very rare. Two patients in the severe dengue group had convulsion after having DSS. There was no correlation between platelet counts and bleeding manifestations in our study. A similar finding was also noted in other studies [11]. Various factors apart from thrombocytopenia lead to bleeding in dengue. They are decreased platelet function, fibrinogen consumption, prolongation of PT/PTT, and vasculopathy [12]. In our study, in the majority of the patients tourniquet test was found to be negative, whereas studies in other countries, especially Southeast Asian countries, report tourniquet test positivity as the commonest bleeding manifestation [13]. Low proportion of positive tourniquet test in Indian studies may be due to the darker skin colour in Indian children [14]. The most consistent finding was hepatomegaly, which was similar to many other studies [10, 11]. Among the various clinical findings hypotension, pleural effusion, and respiratory distress were notable and were analogous to other studies. Leukopenia was seen, which was similar to two other studies [10, 14]. The earliest haematological abnormality is a progressive decline in total WBC count in patients of dengue [2]. Leukopenia was not significantly related with severe dengue cases which were against some results [15]. In our study thrombocytopenia was seen to be more in those with severe dengue [15]. There were a low proportion of children with evidence of haemoconcentration in our study group. The percentage increase in haematocrit is an accurate indicator of vascular permeability and plasma leakage. But it was also reported in previous studies that in some cases the fluid leakage does not achieve a high degree haemoconcentration even if the patient is in shock; this explains our findings. In some DF patients the rise of PCV could have been due to dehydration as a result of poor intake and vomiting [16]. There are no clear-cut guidelines for haemoconcentration in the Indian population. Elevation of SGOT was significantly more compared to SGPT in the present study and is more associated with severity of infection which coincides with others also [14]. SGOT raise more than SGPT in dengue may be due to involvement of myocytes. Value more than 1000 IU/L is seen in severe dengue. Very high levels of SGOT and SGPT indicate severity of the disease along with morbidity and mortality. This differs from the pattern seen in viral hepatitis [17]. In this study one child who had liver enzymes >1000 IU/L succumbed on the second day of hospitalisation in spite of all measures. Rise in PT/aPTT also depicts severity of disease. Ascites and pleural effusion were common presentations, whereas chest X-ray revealed pleural effusion in 25.77%. In USG of the abdomen right sided effusion (15.46%) was most commonly seen which was similar to the previous study [11]. Pleural effusion is more significant in severe dengue. Among types of effusions bilateral pleural effusion is more indicative of severity of the disease. In our study all nonsevere dengue cases recovered. Among the severe dengue cases 12 cases recovered and 1 child expired due to intractable shock.

There was less mortality in the present study group, whereas mortality rate was high in earlier previous studies. This could be due to delay in recognition of epidemic in previous years or delay in seeking medical attention. Case fatality rate (CFR) of the SEAR countries in 2006 was less than 1%. India, Indonesia, Bhutan, and Nepal still have case fatality rates above 1% [18]. Early diagnosis and improved case management of dengue fever are required to bring down CFR to below 1%.

## 5. Conclusion

Dengue is a common disease in this part of the world. It is one of the dreaded fevers for the paediatric age group. The disease has various presentations and features, but early diagnosis and management can decrease case fertility rate significantly. In our study we have enlisted all the typical and atypical presentations, epidemiological data, investigations, and management according to recent WHO guidelines. Severe dengue is very dangerous for children. Lab parameter like raised SGOT is very significant for distinguishing severe disease from nonsevere variety. Pleural effusion is a dominant feature of severe disease. This study will elaborate knowledge about the disease and will improve the outcome.

## Figures and Tables

**Table 1:** Epidemiology.

Parameter	Variables	Numbers	%	Nonsevere dengue	Severe dengue	Stats
Age	<3 yrs	15	15.4%	15	0	Mean age 8.7 yrs
	4–7 yrs	22	22.6%	21	1	
	8–11 yrs	27	27.8%	24	3	
	>11 yrs	33	34%	24	9	

Sex	Male	75	77.3%	66	9	
	Female	22	22.7%	18	4	

Duration of hospitalisation	0–3 days	28	28.8%	27	1	Mean duration 3.8 days
	3–6 days	62	63.9%	57	5	
	>6 days	7	7.2%	0	7	

Day of admission	0–3 days	41	42.2%	38	3	Mean day 3.4 days
	3–6 days	51	52.5%	43	8	
	>6 days	5	5.1%	3	2	

Classification	Undifferentiated fever	31	31.9%			
	DF (with and without warning signs)	53	54.6%			
	Severe dengue fever (DHF)	13	13.4%			

**Table 2:** Investigation.

Investigations	Variations	Nonsevere dengue (*n* = 84)	Severe dengue (*n* = 13)	Total (*n* = 97)	*P* value
TLC	Leukopenia (<4000 cells/mm^3^)	22 (26.19%)	3 (23.07%)	25 (25.77%)	
	Leukocytosis (>11000 cells/mm^3^)	13 (15.47%)	2 (15.38%)	15 (15.46%)	
	Normal TLC (4000–11000/mm^3^)	49 (58.33%)	8 (61.53%)	57 (58.7%)	

Liver enzymes	Rise in SGPT (IU/L)				
	Total	25 (29.76%)	5 (38.46%)	30 (30.92%)	0.5326
	50–200 U	23	1		
	200–1000 U	2	1		
	>1000 U	0	3		
	Rise in SGOT (IU/L)				
	Total	36 (42.85%)	10 (76.92%)	46 (47.42%)	0.0346^*∗*^
	50–200 U	31	1		
	200–1000 U	5	4		
	>1000 U	0	5		

TPC	>100001	66 (78.57%)	4 (30.76%)	70 (72.16%)	
	100000–50001	12 (14.28%)	4 (30.76%)	16 (16.49%)	
	<50000	6 (7.14%)	5 (38.46%)	11 (11.34%)	

Haematocrit	>36.3%	27 (32.14%)	6 (46.15%)	33 (34.02%)	
	<36.3%	57 (67.85%)	7 (53.84%)	64 (65.97%)	

Chest X-ray	Pleural effusion	17 (20.23%)	8 (61.53%)	25 (25.77%)	0.0037^*∗*^
	Right sided effusion	12 (14.28%)	3 (23.07%)	15 (15.46%)	0.4179
	Left sided effusion	4 (4.76%)	0	4 (4.12%)	
	Right + left effusion	1 (1.19%)	5 (38.46%)	6 (6.18%)	0.0001^*∗*^

USG of abdomen	Hepatomegaly	41 (48.80%)	10 (76.92%)	51 (52.57%)	
	Ascites	19 (22.61%)	6 (46.15%)	25 (25.77%)	
	Gall bladder wall edema	0	2 (15.38%)	2 (2.06%)	

Dengue serology	NS1	43 (51.19%)	3 (23.07%)	46 (47.42%)	
	IgM	30 (35.71%)	2 (15.38%)	32 (32.98%)	
	IgG	3 (3.57%)	0	3 (3.09%)	
	IgM & IgG	5 (5.95%)	6 (46.15%)	11 (11.30%)	
	NS1 & IgM	3 (3.57%)	2 (15.38%)	5 (5.14%)	

^*∗*^Significant *P* value.

**Table 3:** Hepatic manifestations.

	Bleeding manifestations			*P* value
	Present	Absent	Total	
Thrombocytopenia				0.3314
Present	11	16	27
Absent	20	50	70
Total	31	66	97
Hepatomegaly				0.2784
Present	17	26	43
Absent	15	39	54
Total	32	65	97
Raised SGOT				0.2808
Present	18	28	46
Absent	14	37	51
Total	32	65	97

**Table 4:** Management.

Management	Nonsevere dengue (*n* = 84)	Severe dengue (*n* = 13)	Total (*n* = 97)
Antipyretics	84	13	97
Intravenous fluids	17 (20.23%)	10 (76.92%)	27 (27.83%)
Platelet transfusion	0	4 (30.76%)	4 (4.12%)
Whole fresh blood transfusion	0	4 (30.76%)	4 (4.12%)
Dopamine	0	4 (30.76%)	4 (4.12%)
Adrenaline	0	1 (7.69%)	1 (1.03%)

## Abbreviations

SGOT: Aspartate transaminase
CFR: Case fatality rate
WHO: World Health Organization
DHF: Dengue haemorrhagic fever
DSS: Dengue shock syndrome
IgM antibody: Immunoglobulin M antibody
IgG antibody: Immunoglobulin G antibody
TLC: Total leukocyte count
TPC: Total platelet count
Hb: Haemoglobin
SGPT: Alanine aminotransferase
aPTT: Activated partial thromboplastin time
PT: Prothrombin time
DF: Dengue fever
PCV: Packed cell volume
USG: Ultrasonography
SEAR: Southeast Asian region.
